# Knowledge of Antimicrobial Resistance and Associated Factors Among Health Professionals at the University of Gondar Specialized Hospital: Institution-Based Cross-Sectional Study

**DOI:** 10.3389/fpubh.2022.790892

**Published:** 2022-03-16

**Authors:** Wudneh Simegn, Baye Dagnew, Berhanemeskel Weldegerima, Henok Dagne

**Affiliations:** ^1^Department of Social and Administrative Pharmacy, School of Pharmacy, University of Gondar, Gondar, Ethiopia; ^2^Department of Human Physiology, School of Medicine, University of Gondar, Gondar, Ethiopia; ^3^Department of Environmental and Occupational Health and Safety, Institute of Public Health, University of Gondar, Gondar, Ethiopia

**Keywords:** knowledge, antimicrobial resistance, health professionals, Hospital, Ethiopia

## Abstract

**Background:**

Antimicrobial resistance is one of the many health challenges worldwide, particularly in resource-limited countries like Ethiopia. Increasing knowledge of health professionals can reduce the occurrence of antimicrobial resistance. In this study, we determined the antimicrobial resistance knowledge and examined the associated factors among the University of Gondar Hospital health professionals.

**Methods:**

An institution-based cross-sectional survey was carried out. The samples were randomly recruited. Statistical analysis was performed by using the statistical package for social sciences (SPSS) version 20 after entering the data using Epidemiological information (Epi-Info). To identify associated factors, the authors executed binary logistic regression and multivariate analysis wherein the statistical significance was decided at *p* < 0.05.

**Results:**

Four hundred and twelve health professionals with ages ranging from 20-60 years and mean age of 29.9 years took part in the study. Fifty-three-point-four percent of participants were males. The majority of the total respondents (84.7%, 95% CI: 80.08–88.30) had good knowledge of antimicrobial resistance. It was found that being male (AOR = 1.94, 95% CI: 1.10, 3.52), a work experience of 6–10 years (AOR = 2.45, 95% CI: 1.28, 4.68), having 30–38working hours per week (AOR = 3.93, 95% CI: 1.38, 5.11), and antibiotic intake (AOR = 3.71, 95% CI: 1.75, 7.87) were significant factors of antimicrobial resistance knowledge.

**Conclusion:**

In the current study, about 84.5% of health professionals had good knowledge of antimicrobial resistance. Reducing working hours per week and increasing the experience of workers are recommended to increase the knowledge on AMR.

## Background

Antimicrobial resistance (AMR) is one of the main global health challenges, particularly in resource-limited countries associated with increased antimicrobial use (1–7). The environment is also increasingly affected by the global spread of clinically relevant antimicrobial resistance (8). It requires international approach and is due to inappropriate use, high load of infectious diseases, poor infection prevention, poor infection control, poor quality drugs, inadequate AMR knowledge, incorrect diagnosis, and absence of laboratory tests for drug susceptibility (3, 4, 9, 10). It is known that treatment of bacterial infections is highly problematic and can create access for the microorganisms to develop resistance (11, 12). As AMR often occurs in seriously ill individuals who often require antibiotic therapy and, in health setups, are in close proximity to each other, there is a possibility of increasing the risk of the emergence and subsequent transmission of resistance within and between patients (13, 14).

The role of health professionals is essential in the rational use of antimicrobials. Potential benefits of awareness of the effects of AMR for health professionals are helpful to prevent any kind of infection, reduce the occurrence of AMR, and promote effective drug use (1, 3, 15, 16). In Ethiopia, there are signs of irrational use of antibiotics in healthcare providers (17). In addition, the previous study identified a problem of information gap on antimicrobial resistance among health professionals (5). The educational background of health professionals and the field of clinical practice were also identified as determinants of the level of awareness of AMR (18).

As it is defined by WHO, rational use of antimicrobials is “the cost-effective use of antimicrobials which maximizes the clinical therapeutic effect, whereas minimizing both drug-related toxicity and the development of AMR (15, 19).” To reduce AMR, different strategies were undertaken, including health education from which rational use of drugs is assumed to be more effective than others (2, 20–22). Several studies reported that rational use of antimicrobials is achieved by developing the prescribing performance and knowledge of the healthcare professionals (4, 23, 24).

Globally, the rate of AMR is growing and becoming a threat to public health, increasing healthcare costs. AMR can result in the reduction of drug efficacy, difficult patient treatment, or increase in cost. At times, it may be impossible to achieve a therapeutic outcome (25). In a year, more that 10 million people worldwide were killed by tuberculosis, malaria, cholera, diarrhea, and pneumonia (13, 26). In Ethiopia, AMR surveillance has been demonstrated in low-resource settings with strong leadership and stakeholder engagement (27).

As there were no previous studies to investigate the knowledge of AMR among health professionals in Ethiopia, particularly in Gondar Comprehensive Specialized Hospital, we undertake this research which could be used as a baseline for researchers to fill the gaps of AMR challenges. Henceforth, this study assessed the knowledge of health professionals on AMR and associated factors in the University of Gondar Comprehensive Specialized Hospital, northwest Ethiopia.

## Methods

### Study Design, Setting, and Period

We conducted a cross-sectional survey among health professionals at the University of Gondar Comprehensive and Specialized Hospital from June to August 2020. The University of Gondar is located in the northwest Ethiopia, 728 km away from Addis Ababa, the Ethiopian capital City. The hospital serves for more than 5 million people in the catchment area. It has about 1,060 health professionals at the time of the study period.

### Source Population

All health professionals working in diverse departments in University of Gondar Comprehensive Specialized Hospital were the source of the population.

### Study Population

Health professionals who are in the department of nursing, pharmacy, medicine, laboratory, and midwifery, all working with diverse departments in the University of Gondar Comprehensive Specialized Hospital were the included in the study population.

### Inclusion and Exclusion Criteria

All health professionals (in department of nursing, pharmacy, medicine, laboratory, and midwifery) working at the University of Gondar Comprehensive and Specialized Hospital were included. Some health professionals were excluded due to low contribution to the prevention of AMR. Health professionals who had <6 months of work experience and were on annual leave were also excluded.

### Sample Size Calculation and Sampling Technique

The sample size was calculated using single population proportion formula with assumptions about the proportion = 0.5, 95% uncertainty interval, and margin of error (d) = 5%. By adding a non-response rate of 10%, the final sample size was 423. We used a simple random sampling technique for the selection of study participants to be included.

### Data Collection Procedure

**Figure 1:**
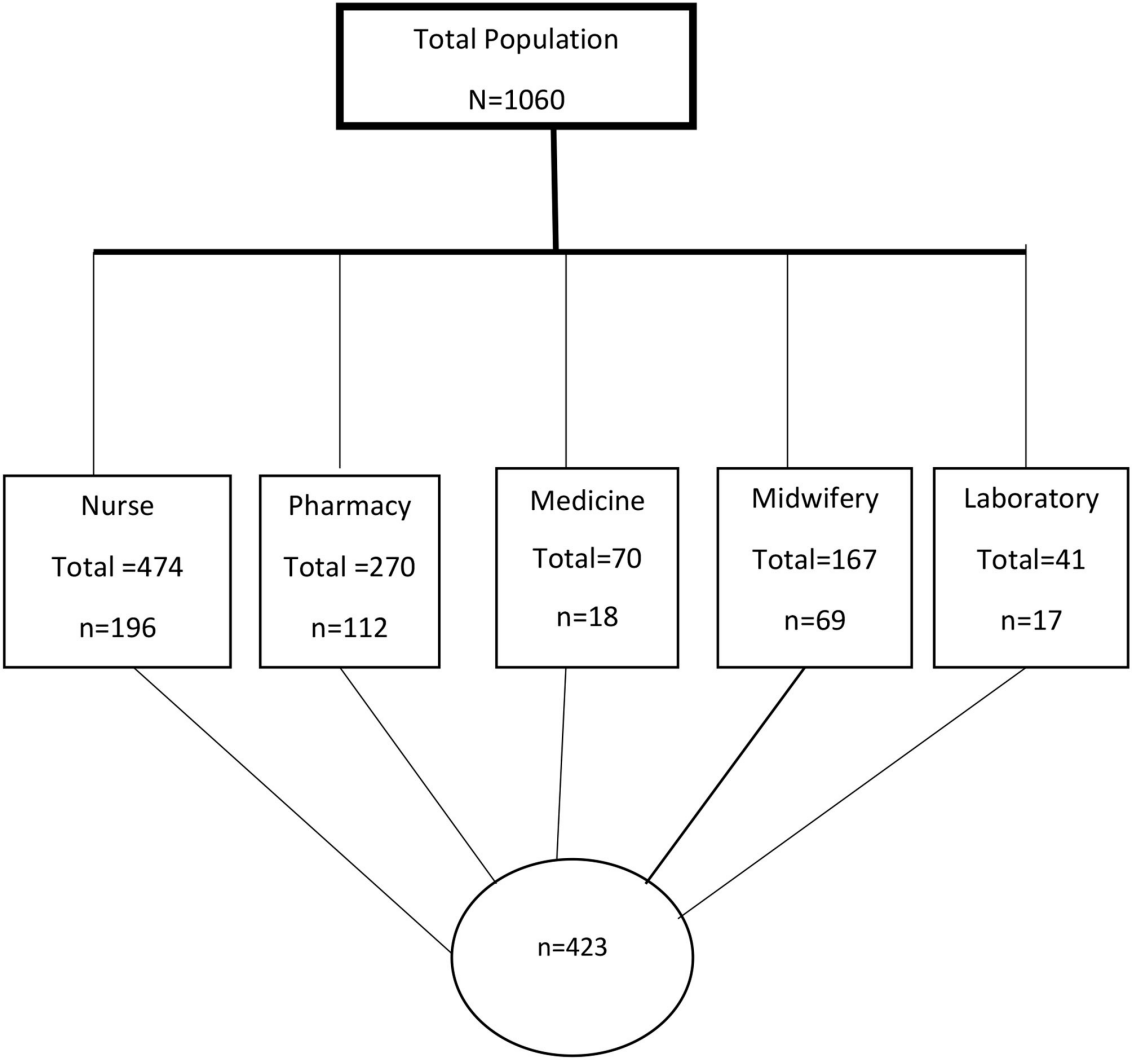
Flow chart of study participants for knowledge of antimicrobial resistance among health professionals at the University of Gondar Comprehensive and Specialized Hospital, Gondar, Northwest Ethiopia, 2020.

We developed a pretested semi-structured, self-administered questionnaire from previously published studies (4, 7, 28) with some adjustments based on the study subjects and set up. Sociodemographic characteristics and knowledge of AMR of health professionals were components of the data collection tool (eight true/false questions) that was mainly designed to identify varies aspects of participants' knowledge about AMR. Internal consistency was checked using the Cronbach's alpha, which was 0.89, i.e., acceptable. Questionnaires were distributed to health professionals working both night and daytime shifts. Four BSc Nurses were recruited to distribute, facilitate the collection, and return the questionnaires (Figure 1).

### Data Measurement Techniques

The measuring techniques was, as per stated in the WHO, the level of measurement of antimicrobial resistance of communities (29). Each participant was subjected to answer eight questions, whereby a score of one is rewarded for a correct answer and zero for an incorrect answer. A score below 75% was considered to have fair and low (totally poor) AMR knowledge, while those whose scores ≥75% were considered to have good AMR knowledge. These cutoff points were based on a previous study from Nigeria which was based on WHO criteria (30).

### Study Variables

The dependent variable was knowledge of AMR, while potential independent variables were sex, age, marital status, education level, duration of work experience, setting (working section), profession, working hours per week, presence of work stress, and self- medication practice.

### Operational Definitions

#### Work Stress

Emotional responses that occur when the requirements of the job do not match the capabilities, resources, or needs of the worker. Respondents were asked whether they face symptoms of work stress within a single question. They were to answer as none, low, medium, or high based on their subjective opinion.

#### Workload

The high amount of work or working time given or assigned to do per day. Respondents were asked to answer as none, low, medium, or high based on their subjective opinion on their workload in the department.

#### Self-medication Practice

The use of medication or drugs to treat self-diagnosed disorders or symptoms by purchasing at a community pharmacy without the prescription of other health professionals and without following appropriate diagnosis by the health practitioner team. Respondents were asked to answer “yes” if they had ever used drugs/pharmaceutical products for themselves without prescription and “no” if otherwise.

#### Knowledge of Over the Counter (OTC) Drug Classification

The knowledge of participants in classifying drugs/pharmaceutical products sold OTC (without prescription) based on safety, effectiveness, and appropriateness, i.e., assurance that the patient can manage without supervision (e.g., paracetamol, ibuprofen, and vitamin complexes). Respondents were asked to answer “yes” for this question if they knew this classification and “no” if otherwise.

### Statistical Analysis

We used Epi-info 7.1 for data entry before exporting into SPSS version 20 for computing, recoding, and statistical analysis. Mean with standard deviation (SD) and frequency were calculated. Binary logistic regression was fitted to identify associated factors of knowledge of AMR. After checking for bivariable association (at *p* < 0.2), we performed a multivariable analysis using odds ratio with its 95% CI to describe the strength of association, and a *p* < 0.05 to ascertain the statistical significance to describe the association between exposure variables with the outcome variable (knowledge of AMR).

### Data Quality Control

As quality measure, we conducted a pretest among 20 individuals at the Felege Hiwot Specialized Hospital. Adjustment of question order, addition of some sociodemographic variable, and editorial amendments were done to assure face validity of the assessment tool after the pre-test. Training about the purpose and techniques of data facilitation was delivered to the data collection facilitators. Amendments were made based on the pretest results.

## Results

### Socio Demographic Character of Study Participants

A total of 412 participants (220 were males) took part in the study. There was a 97.4% response rate. The participants' mean age was 29.9 years (±5.43, range = 20–60). The majority of participants (82.3%) held a Bachelor's degree. From the participants, majority (47.6%) were Nurse professionals, followed by pharmacy professionals (27.2%) and Midwifery (16.7%). Of the total participants, 58.3% had 2–5 years of work experience and 39.1% of respondents work for 39–43 h a week (Table 1).

**Table 1:** Sociodemographic characteristics of study participants in University of Gondar Comprehensive Specialized Hospital, Gondar, Ethiopia, 2020 (*n* = 412).

**Variable**	**Categories**	**Frequency**	**Percent**
Marital status	Married	226	54.9
	Unmarried	186	45.1
Sex	Female	192	46.6
	Male	220	53.4
Age in years	Mean = 29.92(±5.43) Min = 20	Max = 60	
	20–25	69	16.7
	26–28	133	32.3
	29–31	91	22.1
	32–60	119	28.9
Education level	Diploma	26	6.3
	Bachelor degree	339	82.3
	Masters and above	47	11.4
Work experience	1–5	240	58.3
in years	6–10	143	34.7
	11–37	29	7.0
Profession	Nurse	196	47.6
	Pharmacy	112	27.2
	Medicine	18	4.4
	Midwifery	69	16.7
	Laboratory	17	4.1
Working hours per	30–38	45	10.9
week	39–43	161	39.1
	44–55	97	23.5
	56–110	109	26.5
Work stress out of	Very low	51	12.4
work	Low	84	20.4
	Medium	160	38.8
	High	117	28.4
Average number contacted per day	4–9	100	24.3
of patients	10–14	85	20.6
contacted per day	15–29	59	14.3
	30–200	16	40.8
Work load	None	32	7.8
	Low	43	10.4
	Medium	165	40.0
	High	172	41.7

### Medication Practice and Knowledge of AMR

From a total of 412 respondents, 349 health professionals (84.7%, 95% CI: 80.08–88.30) had good knowledge of AMR. Three hundred and forty-three (83.3%) health professionals reported that they have used antibiotics for self-treatment in their lifetime. Among these, 183 (53.4%) participants have taken antibiotics within a year. Only a few participants (17.2%) gained training regarding AMR. Of the participants, 225 (54.6%) participants reported to practice self-medication in their life (Table 2).

**Table 2:** Medication Practice and knowledge of antimicrobial resistance by Health Professionalsin University of Gondar Comprehensive Specialized Hospital, Gondar, Ethiopia, 2020 (*n* = 412).

**Variable**	**Categories**	**Frequency**	**Percent**
know OTC drugs classification	Yes	311	75.5
	No	101	24.5
Ever taken antibiotics	Yes	343	83.3
	No	69	16.7
Used antibiotics within a year	Yes	183	44.4
	No	229	55.6
Self-medication Practice	Yes	225	54.6
	No	187	45.4
Ever had training about AMR	Yes	71	17.2
	No	341	82.8
Knowledge of antimicrobial resistance	Good	349	84.7
	Fair	46	11.2
	Poor	17	4.1

### Associated Factors for Knowledge of AMR

In the bivariable analysis, sex, work experience, working hours per week, work stress, knowledge of over the counter drugs, use of antibiotics, and self-medication practice were associated with knowledge of AMR. Finally, we found that knowledge of AMR was significantly associated with being male (AOR = 1.94, 95% CI: 1.10, 3.52), 6–10 years of work experience (AOR = 2.45, 95% CI: 1.28, 4.68), 30–38 working hours per week (AOR = 3.93, 95% CI: 1.38, 5.11.12), and antibiotics intake (AOR = 3.71, 95% CI: 1.75, 7.87) (Table 3).

**Table 3:** Associated factors for knowledge of antimicrobial resistance among Health professionals at University of Gondar Comprehensive Specialized Hospital, Gondar, northwest Ethiopia, 2020 (*n* = 412).

**Variables**	**Categories**	**Knowledge**		**COR (95% CI)**	**AOR (95% CI)**
		**Good (%)**	**Poor (%)**		
Sex	Female	154 (44.1)	38 (60.3)	1	1
	Male	196 (55.9)	25 (39.7)	1.93 (1.11, 3.33)	1.94 (1.10, 3.52)^*^
Work experience	1–5	216 (61.9)	24 (38.1)	1	1
	6–10	112 (32.1)	31 (49.2)	2.49 (1.40, 4.45)	2.45 (1.28, 4.68)^**^
	11–37	21 (6.0)	8 (12.7)	3.43 (1.37, 8.58)	3.94 (1.43, 10.80)^**^
Working hours per week	30–38	33 (9.5)	12 (19.0)	3.24 (1.31, 8.03)	3.93 (1.38, 5.11)^*^
	39–43	139 (39.8)	22 (34.9)	1.41 (0.65, 3.04)	1.15 (0.49, 2.72)
	44–55	79 (22.6)	18 (28.6)	2.03 (0.91, 4.55)	1.95 (0.82, 4.64)
	56–110	98 (28.1)	11 (17.5)	1	1
Work stress	Very low	23 (6.6)	9 (14.3)	1	1
	Low	32 (9.2)	11 (17.5)	2.97 (1.21, 7.32)	3.01 (1.06, 8.58)
	Medium	142 (40.7)	23 (36.5)	2.61 (1.14, 5.98)	1.74 (0.67, 4.56)
	High	152 (43.6)	20 (31.7)	1.23 (0.65, 2.34)	1.08 (0.54, 2.16)
know OTC classification of drugs	Yes	271 (77.7)	40 (63.5)	2.00 (1.13, 3.54)	1.06 (0.52, 2.23)
	No	78 (22.3)	23 (36.5)	1	1
Ever taken antibiotics	Yes	304 (87.1)	39 (61.9)	4.16 (2.29, 5.55)	3.71 (1.75, 7.87)^**^
	No	45 (12.9)	24 (38.1)	1	1
Self-medication practice	Yes	196 (56.4)	28 (44.4)	1.62 (0.94, 2.78)	1.42 (0.76, 2.65)
	No	152 (43.6)	35 (55.6)	1	1

*Hosmer and Lemeshow good-ness of fit p = 0.86*

* *p < 0.05*

** *p < 0.01*.

## Discussion

As AMR is a major global health problem and health professionals are key stakeholders in the prevention and control of AMR during prescribing and dispensing antibiotics and related activities, the authors conducted the cross-sectional study that assessed the health professionals' knowledge on AMR (5, 31). In line with this, the current study aimed to assess the knowledge and associated factors of health professionals about AMR in the University of Gondar Comprehensive and Specialized Hospital.

In this study, the respondents' knowledge on AMR was 84.7% (95% CI: 80.08–88.30), which is in line with other studies (10, 31–33). However, this study showed a higher value of knowledge than some other studies conducted in the country (5, 34, 35). This discrepancy could be accounted for by sample size, methodology, literacy, in service training, study setting, and sociocultural factors.

In this study, factors such as being of the male sex, work experience, working hours per week, and antibiotic intake were found to be significantly associated with knowledge on AMR. Compared to females, males were 1.93 times more likely to have better knowledge on AMR in the current study. However, the statistical differences between sex was not observed (*p* > 0.05) in a previous study conducted in Dire Dawa, Ethiopia (34). These discrepancies might be due to other latent factors that mediate knowledge rather than sex disparity.

Health professionals who had more than 11 years of work experience had 3.43 times better knowledge of AMR compared to those health professionals having only 1–5 years of work experience. This is not surprising as experience can improve knowledge through different exposures through time. This improvement can also be facilitated by working with seniors (36). In addition respondents having few working hours per week (30–38 h) showed higher knowledge of AMR (3.23 odds) compared with respondents having high working hours per week (56–110 h). This might be due to the presence of free time for reading updated science. In the current study, health professionals who had experience in taking antibiotics were 3.7-fold more likely to have better knowledge of AMR. This might be due to how health professionals may read more about the antibiotics during use to can gain better information about AMR. Our findings revealed that respondents with self-reported awareness of OTC drugs classification were about 2 times more likely to have knowledge of AMR. In addition, healthcare professionals with experience of self-medication practice were more likely to have about 1.5 times better knowledge of AMR.

However, other studies showed that AMR is mostly developed by self-medication (5, 31, 34). In the current study, awareness of OTC drugs classification and self-medication were not significantly associated with AMR knowledge (*p* > 0.05) even though they were the candidate variables for multivariate regression (*p* < 0.2).

## Limitation of the Study

Even though this study used a large sample size and was able to find possible contributing factors for health professionals' knowledge of AMR, it also had limitations including recall and social desirability bias. In addition, the cause-effect association cannot be elucidated by using a cross-sectional survey.

## Conclusion

The current study showed that 84.5% of health professionals had good knowledge of AMR. Male sex, high work experience, low working hours per week, and history of antibiotic intake were found to be contributing factors with good knowledge of AMR. Reducing working hours per week is recommended to increase the knowledge of AMR.

## Data Availability Statement

The raw data supporting the conclusions of this article will be made available by the authors, without undue reservation.

## Ethics Statement

Ethical approval was gained from the Ethical Committee of the School of Pharmacy, University of Gondar with ethical review protocol number: SOP/559/2019. All participants were informed about the purpose of the study. Written informed consent was obtained from each participant to assure their willingness of participation and no identifiers were listed in the questionnaire to make it confidential.

## Author Contributions

WS involved in the proposal development, analysis, and manuscript writing. BD, HD, and BW participated in statistical analysis and manuscript preparation. All authors reviewed and approved the final manuscript.

## Conflict of Interest

The authors declare that the research was conducted in the absence of any commercial or financial relationships that could be construed as a potential conflict of interest. The reviewers AM and AS declared a shared affiliation with the authors, to the handling editor at the time of review.

## Publisher's Note

All claims expressed in this article are solely those of the authors and do not necessarily represent those of their affiliated organizations, or those of the publisher, the editors and the reviewers. Any product that may be evaluated in this article, or claim that may be made by its manufacturer, is not guaranteed or endorsed by the publisher.

## Abbreviations

AMR: Antimicrobial resistance
HCP: Healthcare professionals
OTC: Over the counter
WHO: World health organization.
